# Integrated children’s clinic care (ICCC) versus a self-directed care pathway for children with a chronic health condition: a multi-centre randomised controlled trial study protocol

**DOI:** 10.1186/s12887-018-1034-x

**Published:** 2018-02-19

**Authors:** Thuy Thanh Frakking, John Waugh, Hsien-Jin Teoh, Doug Shelton, Susan Moloney, Donna Ward, Michael David, Matthew Barber, Hannah Carter, Sharon Mickan, Kelly Weir

**Affiliations:** 1 Research Development Unit, Caboolture Hospital, Queensland Health, McKean St, Caboolture, QLD 4510 Australia; 2 School of Health & Rehabilitation Sciences, The University of Queensland, St Lucia, QLD Australia; 3 School of Clinical Medicine, The University of Queensland, St Lucia, QLD 4067 Australia; 4 Department of Paediatrics, Caboolture Hospital, Queensland Health, McKean St, Caboolture, QLD 4510 Australia; 5 Department of Community Child Health, Gold Coast University Hospital, Queensland Health, Southport, QLD 4215 Australia; 6 Department of Paediatrics, Gold Coast University Hospital, Queensland Health, Southport, QLD 4215 Australia; 7 School of Population Health, The University of Queensland, Herston, QLD 4006 Australia; 8 Ningi Doctors, 1421-1423 Bribie Island Rd, Ningi, QLD 4511 Australia; 9 Brisbane North Primary Healthcare Network, Lutwyche, QLD 4030 Australia; 10 Australian Centre for Health Services Innovation, Queensland University of Technology, Kelvin Grove, QLD 4059 Australia; 11 School of Allied Health Sciences & Menzies Health Institute Queensland, Griffith University, Gold Coast, QLD 4222 Australia; 12 Clinical Governance, Education & Research, Gold Coast University Hospital, 1 Hospital Boulevard, Southport, QLD 4215 Australia

**Keywords:** Integrated care, Care coordination, Chronic health condition, Paediatrics, Randomised controlled trial, Logic model

## Abstract

**Background:**

Children with chronic health conditions have better health-related outcomes when their care is managed in a personalised and coordinated way. However, increased demand on Australian ambulatory care hospital services has led to longer waitlist times to access specialists and appropriate intervention services; placing vulnerable children at increased risk of poorer short-term (e.g. social difficulties) and long-term (e.g. convictions) health and social outcomes. Traditional approaches to increasing frequency and service of delivery are expensive and can have minimal impact on caregiver burden. A community based service-integration approach, rather than self-directed care is proposed as increased service linkages are more likely to occur and improve the health outcomes of children with a chronic health condition.

**Methods:**

An open, unblinded, multi-centre randomised controlled trial in two Australian public hospitals. 112 children (0–16 years) fulfilling the inclusion criteria will be randomised to one of two clinical pathways for management of their chronic health condition: (1) integrated children’s care clinic (ICCC) or (2) self-directed care pathway. All children and caregivers will be interviewed at 1 week, and 3, 6 and 12 month time intervals. Primary outcome measures include the Pediatric Quality of Life (PedQOL) questionnaire, Subjective Units of Distress Scale, Child Behaviour Checklist (CBCL) and Rotter’s Locus of Control Scale. Secondary outcome measures include the total number of medical appointments, school days missed and quantity of services accessed. Our main objectives are to determine if the ICCC results in better health and economics outcomes compared to the self-directed care pathway.

**Discussion:**

The success of a health systems approach needs to be balanced against clinical, mortality and cost-effectiveness data for long-term sustainability within a publicly funded health system. A clinical pathway that is sustainable, cost-effective, provides efficient evidence-based care and improves the quality of life outcomes for children with chronic health conditions has the potential to reduce waitlist times, improve access to health services, increase consumer satisfaction; and prevent costs associated with poorly managed chronic health conditions into adulthood. This study will be the first to provide clinical and health economics data on an integrated care pathway for the management of chronic health conditions in children. On a broader scale, results from this study will help guide care coordination frameworks for children with chronic health conditions; particularly with the introduction and implementation of a National Disability Insurance Scheme (NDIS) across Australia.

**Trial registration:**

Australia and New Zealand Clinical Trials Register (ANZCTR) ACTRN12617001188325. Registered: 14th August, 2017.

**Electronic supplementary material:**

The online version of this article (10.1186/s12887-018-1034-x) contains supplementary material, which is available to authorized users.

## Background

Chronic health conditions such as Attention Deficit Hyperactivity Disorder (ADHD) and intellectual impairment (II), have prevalence rates of approximately 5% [1] and 1–3% [2] in the population, respectively. Such a demand has led to longer waitlist times to see a specialist and access appropriate services within the public health sector in Australia. In addition, poorer short-term (e.g. increased risk of mental health, social difficulties) and long-term (e.g. convictions, arrests) outcomes have been reported for vulnerable children with multiple risk factors (i.e. income, education, social support) [3]. Traditional approaches to increasing frequency and service of healthcare delivery are expensive and have been shown to have minimal impact on caregiver burden [4] and changes to caregiver perception of their reduced influence over their current circumstances, including behavioural issues and overall well-being of their children [5, 6]. Research has shown that caregivers who have higher levels of external locus-of-control may be more likely to have issues with taking action to influence their children’s behaviours [5]. There is also emerging literature that suggests that locus-of-control may act as a mediator in the relationship between parenting and the caregivers’ mental health [6]. These findings suggest that a caregivers’ locus-of-control may influence their ability to undertake services and therapies which require regular and active involvement in the management of their child’s chronic health condition and well-being. Given this context, a different approach to health care systems in the management of chronic health condition in children which includes caregiver/family involvement will likely improve both child and caregiver health and social outcomes [7].

Children with chronic health conditions have better health-related outcomes when their care is managed in a coordinated way [8]. Specifically for children with ADHD there are improvements in functional outcomes when families receive more personalized and coordinated care [9] .The Chronic Care Model [10] is the most well-known model used to address healthcare systems in the management of chronic health conditions. Improved quality of life, clinical outcomes and a reduction in health costs have been seen in adults with chronic health conditions (e.g. diabetes, heart disease, chronic obstructive pulmonary disease) when the Chronic Care Model [10] was utilised. Different components (e.g. care coordination) of the Chronic Care Model [10] have been successfully used in the management of children with asthma and ADHD [1, 11, 12]. However, further research utilising the Chronic Care Model [10] to address health care outcomes in vulnerable children with other types of developmental chronic health conditions is required.

The success of a health systems approach needs to be balanced against clinical, mortality and cost-effectiveness data for long-term sustainability within a publicly funded health system. Similar to features of the Chronic Care Model, [10] community based service-integration approaches have been shown to reduce neonatal and maternal morbidity in developing countries [13] and increase the number of service linkages for low income families in a developing country. [14] Features of community based service integration approaches include health worker visits in the community, training of community staff, health promotion, availability of resources to link into existing community services and the allocation of a case manager to the child and family. However, despite reported success in reducing morbidity and increasing access for vulnerable children in developing or low-income families, cost-effectiveness data on the community service-integration models used has not been included in study designs. Nevertheless, reduced health care system costs (e.g. reduced inpatient days, reduced out of pocket expenses) has been shown when care coordination between community and tertiary care providers is provided to children with medically complex conditions [15]. No such information is available for children with chronic health conditions. Further short and long-term health economics information, which includes financial impacts within the school, family and health care environments are required, particularly for children with chronic health conditions. Such health economics information is a gap in current literature and needs to be addressed to ensure publicly funded community service-integrated models of care are cost-effective and sustainable in the long-term.

Based on available literature and increasing financial pressures to cost-effectively sustain a public health care system for children with chronic health conditions, an integrated health care pathway which incorporates the care coordination features of the Chronic Care Model [10] and includes caregiver involvement as an essential component has the potential to improve health and social benefits in an already at risk population. The development of an Integrated Children’s Clinic Care (ICCC) pathway which incorporates features of the Chronic Care Model [10] and involvement of caregivers was developed to improve health and social benefits for children with chronic health conditions. This protocol paper outlines a randomised control trial evaluating the cost-effectiveness of the ICCC pathway. The ICCC centres around an allied health liaison officer (AHLO) facilitating key components of the Chronic Care Model [10] in primary, community and acute care facilities across two Australian health districts of varying socioeconomic status’.

## Methods

### Aims

This randomised controlled trial protocol investigates the cost-effectiveness of an integrated care pathway between hospital and primary care partnerships in improving health and social outcomes in children with chronic health conditions. More specifically, our objectives are to: (1) determine if the ICCC results in better health outcomes for children with chronic health conditions, compared to a self-directed care pathway; and (2) determine the cost-effectiveness of the ICCC using health economics data. We hypothesize that: (a) Children who access the ICCC will have improved quality of life and behavioural scores than children who access the self-directed care pathway; and (b) the ICCC pathway is more cost-effective than the self-directed care pathway.

### Study design

Open, unblinded, multi-site randomised controlled trial in two Australian public hospitals - Caboolture Hospital and Gold Coast University Hospital (GCUH) Paediatric Outpatient Clinics. The Caboolture region has a social economic index for areas (SEIFA) equivalent to 0.1%, while Gold Coast region has a SEIFA ranging from 0.01 to 0.09%.

### Participants

Children aged 0 to16 years with a newly diagnosed chronic heath condition. Inclusion criteria:

Children aged 0 to 16 years who are seen by Paediatrician at Caboolture Hospital or GCUH and newly diagnosed with a chronic health condition where community based health or family support services are part of the management plan. Chronic health conditions are expected to last more than 6 months and to produce consequences that impact on the child’s quality of life [16]. Examples of chronic health conditions include (but are not limited to): Autism Spectrum Disorder (ASD), Attention Deficit Hyperactivity Disorder (ADHD), Intellectual Impairment (II), Specific Language Impairment (SLI), Oppositional Defiance Disorder (ODD), Fetal Alcohol Spectrum Disorder (FASD), Cerebral Palsy (CP).

Exclusion criteria: Children with acute medical conditions requiring urgent intervention where community follow-up is deemed inappropriate by the treating Paediatrician and /or children with a chronic medical condition primarily managed by medical consultation alone and those conditions where hospital based multidisciplinary teams provide coordinated care. Examples of excluded chronic health conditions include: cancer, cystic fibrosis, asthma and epilepsy.

### Recruitment

Children attending paediatric outpatient clinics with the Paediatrician at Caboolture Hospital or GCUH will be approached to participate in the study at the conclusion of their medical appointment by the Allied Health Liaison Officer (AHLO). The caregivers will be provided with a parent information sheet (Additional file 1) and study brochure and informed consent will be gained at this time with or as close as possible to this time. The researcher will verbally explain the caregiver information sheet, study brochure and consent form for all caregivers of eligible participants. If parents/caregivers consent for their family to be part of the study, then they will be asked to sign the consent form and initial and date each page of the caregiver information sheet and study brochure to acknowledge that they have understood the study requirements. When necessary, particularly for children of non-English speaking backgrounds, interpreter services will be used to aid in providing informed consent.

For caregivers who identify with literacy issues, the AHLO will ensure extra time is used to explain the study and obtain informed consent. [17] Before finalising consent, the AHLO will be required use the “teach back” method and ask the caregiver to explain in their own words what the research study is asking them to do, including risks and processes involved [18].

It is anticipated that a high proportion of caregivers will consent to be a part of this trial; however, we anticipate a high drop-out rate due to the length of the trial, anticipated social issues and previously documented high drop-out rates in a similar study [14]. A high attrition rate (approximately 50%) has been factored into the sample size calculations below.

### Randomisation

A randomisation list, created by an independent biostatistician will be used. Block size permutation of *n* = 7 will be utilized to ensure equal distribution of participants into each pathway. Assessment allocation (ICCC versus self-directed care pathway) will be concealed in sequentially numbered opaque envelopes and assigned to enrolled children immediately after informed consent is gained to participate in the research trial by the AHLO. The envelope will be opened in front of the caregiver and child. This would mean that the caregiver, child and AHLO involved in the trial will not be blinded to group assignment. Blinding in this trial is not feasible due to the nature of the ICCC pathway (i.e. caregivers will expect a multidisciplinary appointment with a General Practitioner).

### Data collection

**Fig. 1:**
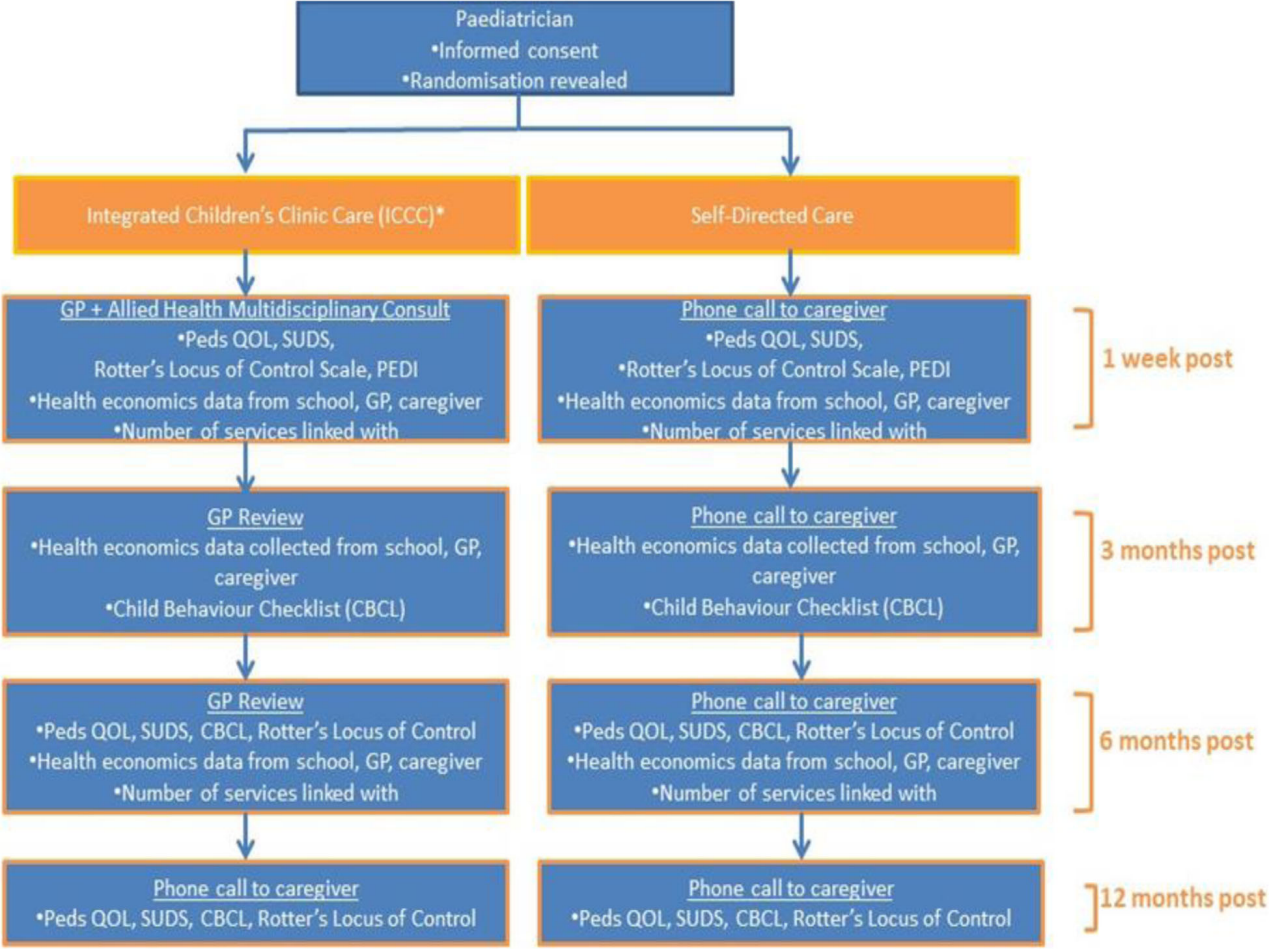
Flow diagram of randomised control trial. * Allied Health Liaison Officer (AHLO) helps family navigate: school (e.g. individual educational plan, supports), allied health (e.g. public/private), community resources (e.g. neighbourhood centre, Moreton Bay libraries), Centrelink (e.g. allowances) in conjunction with GP, Paediatrician and/or other relevant staff in community. Abbreviations: ICCC - Integrated Children’s Clinic Care, GP - General Practitioner, Peds QOL – Paediatric Quality of Life Scale, SUDS – Subjective Units of Distress Scale, PEDI – Pediatric Evaluation of Disability Inventory, CBCL - Child Behaviour Checklist, AHLO – Allied Health Liaison Officer

An outline of the trial can be found in Fig. 1. A text message will be sent to the caregiver 24 h before a planned phone review to serve as a reminder and/or provide an opportunity for the caregiver to negotiate an appropriate time/date for the phone review. It is anticipated that a text message will facilitate increased participation and reduce attrition rates over the period of the study. A maximum of 3 phone attempts will be made at any review time point.

#### Integrated Children’s care clinic (ICCC) pathway

At one week post Paediatrician appointment, the AHLO will make contact with the child’s General Practitioner (GP) to arrange a multidisciplinary long face-to-face meeting to help facilitate recommendations by the Paediatrician. This may include access to community allied health services. The child and caregiver will be seen by the GP at the conclusion of the multidisciplinary meeting for a consult. The AHLO will complete the following with the caregiver/child post GP consult (based on 7 day period):

- ○ Ask how many services the child is currently accessing
- ○ Number of days caregiver missed employment
- ○ Collect baseline demographics data (family structure, primary carer education, primary carer employment status and mental health status). Please note: if caregiver reports emotional distress, then AHLO will recommend for caregiver to see their GP for further assistance.
- ○ Complete Pediatric Evaluation of Disability Inventory (PEDI) if child is between 6 months to 7 years.

**Fig. 2:**
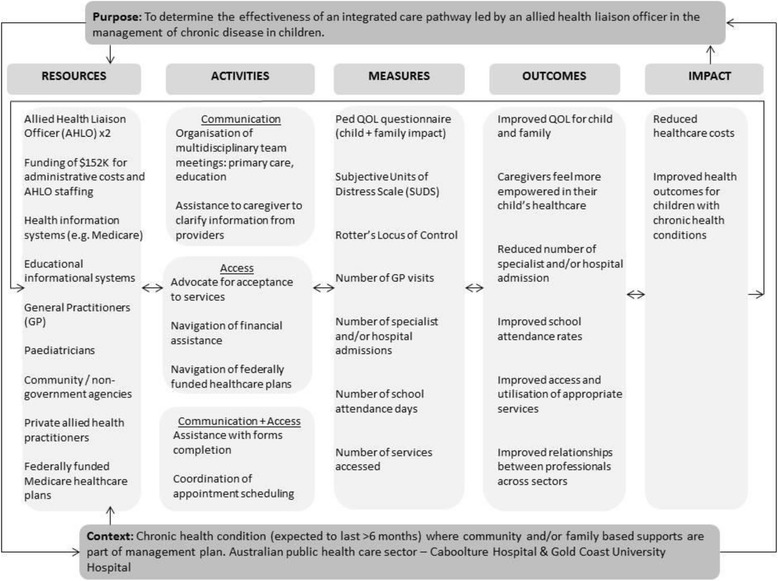
Logic model outlining complex interactions of processes for proposed randomised control trial in the evaluation of an Allied Health Liaison Officer (AHLO). Abbreviations: AHLO – Allied Health Liaison Officer, GP – General Practitioners, Ped QOL – Pediatric Quality of Life, SUDS – Subjective Units of Distress Scale

The AHLO will contact Education Queensland to request for number of school attendance and absent days, including any formal suspensions (if applicable). The AHLO will go through the following checklist to ensure completion. Liaison with other professionals and agencies will occur, as appropriate. Each process used and outcome will be documented to guide process evaluation for the AHLO role [19]. Please refer to Fig. 2, which outlines the logic model used to guide evaluation of the role of the AHLO in this trial. The AHLO will also submit Medicare forms to obtain information on the: number of GP visits, hospital admissions and specialist appointments for the preceding 7 days. Medical Students from each hospital will call the primary caregiver to complete the Pediatric Quality of Life (Ped QOL) Child and Family Impact Modules, [20] Subjective Units of Distress Scale (SUDS) and Locus of Control Questionnaire, and the opportunity to describe any other issues or concerns that they may have regarding their child.

At 3 months (+/− 14 days) post Paediatrician appointment, the AHLO will contact Education Queensland to request for number of school attendance and absent days, including any formal suspensions (if applicable). The AHLO will submit Medicare forms to obtain information on the number of GP visits, hospital admissions and specialist appointments for the preceeding timeframe between 1 week and 3 months post Paediatrician appointment. The AHLO will also help arrange a GP long face-to-face consultation appointment to review the child to check on progress of management plan in relation to the child’s chronic health condition. Finally, the AHLO will complete the following with the caregiver/child post GP consult: ask how many services the child is currently accessing, ask the caregiver how many days they had missed employment and complete the Child Behaviour Checklist (CBCL), [21] and further information that the caregiver would like to add about what issues the child and/or family might be encountering related to access to services/support.

At 6 months (+/− 14 days) post Paediatrician appointment, the AHLO will contact Education Queensland to request for the number of school attendance and absent days, including any formal suspensions (if applicable). The AHLO will also submit Medicare forms to obtain information on the number of GP visits, hospital admissions and specialist appointments for the preceeding timeframe between 3 months to 6 months post Paediatrician appointment. Finally, the AHLO will help arrange a GP long face-to-face consultation appointment to review the child. The AHLO will also call the caregiver to: ask how many services the child is currently accessing, ask how many days the caregiver missed employment and complete the CBCL [21]. Medical Students from each hospital will also call the primary caregiver to complete: Peds QOL Child and Family Impact Modules, [20] SUDS and Locus of Control Questionnaire.

At 12 months (+/− 14 days) post Paediatrician appointment, medical students from each hospital will call the primary caregiver to complete: Peds QOL Child and Family Impact Modules, [20] SUDS and Locus of Control Questionnaire.

#### Self-directed care pathway

The procedure for self-directed care pathway is similar to ICCC, except the participants will not have access to an AHLO to: (a) coordinate GP appointments at 1 week, 3 and 6 months post Paediatrician appointment, and (b) assist with any communication and access issues that arise during the duration of the study.

#### Outcome measures

Primary outcome measures taken at 1 week, 3, 6, 12 months include**:** PedQOL child module (Score 0 to 100; parent or child completed), [20] PedQOL family impact module (Score 0 to 100; parent completed), [20] SUDS (Score 0 to 100; parent completed), CBCL (Percentiles, parent completed) [21] and Rotter’s Locus of Control Scale (Score 0 to 23, parent completed) [22].

Secondary outcome measures taken at 1 week, 3, 6, 12 months include the number of**:** GP visits, hospital admissions**,** specialist appointments**,** absent school days, caregiver missed employment days, school suspensions including duration in days and services currently accessed at the time.

### Sample size and statistical power

We plan for a total sample size of 112 children (80% power to detect a mean effect difference of 15 between groups on the quality of life scale) at 0.05 significance level. This sample size has been adjusted for an anticipated high attrition rate of 50%. A 2-sample test of proportions will be used to compare baseline characteristics of both groups.

### Statistical analyses

All analyses will be conducted using an “intention to treat” (ITT) analysis where all subjects will be compared in the groups which they were originally assigned (regardless of withdrawal or lost to follow-up). For our primary objective, Mann Whitney U test will be used to determine if differences in quality of life measures exist between the ICCC versus self-directed care groups.

For our secondary objective, we will perform univariable analysis to determine which health economic parameters (e.g. missed school days, number of hospital admissions) are related to higher quality of life scores. Stepwise regression will then examine the various combinations via of health economic parameters to generate area under receiver operating curves (aROC) to determine a clinical prediction index for higher quality of life scores. Parameters chosen will be based on using factors that were significant plus those with *P* < .25 level in the univariable analysis and other variables known to have a strong association with poorer health outcomes (e.g. multiple co-morbidities) within the literature. An aROC of ≥0.75 will be considered a clinically relevant cut-off score [23].

As this study is registered on the ANZCTR and occurring within Metro North Hospital and Health Service, the trial may be randomly audited by an independent study monitor at any timepoint in the study. A data monitoring committee will not be set up for the purposes of this study, as it is unblinded and not comparing rates of mortality or major morbidity.

### Safety considerations/patient safety

The study will be conducted in full conformance with principles of the Declaration of Helsinki [24] and Good Clinical Practice (GCP) [24]. Children randomised to the self-directed pathway will be allowed to change to the ICCC pathway after 6 months, if they wish. However, their recorded data at 12 months will not be used in the final analyses. Identified information will only be shared and viewed by investigators involved in patient care or data collection. De-identified information will only be seen by investigators on the project. Data will be stored in locked cabinet at Caboolture Hospital for 5 years post study completion, as per National Health and Medical Research Council (NHMRC) guidelines.

For caregivers who identify with literacy issues, the AHLO will ensure extra time is used to explain the study and obtain informed consent [17]. Before finalising consent, the AHLO will be required use the “teach back” method and ask the caregiver to explain in their own words what the research study is asking them to do, including risks and processes involved [18]. For children age ≥ 12 years, assent will be obtained for participation in the study in conjunction with caregiver consent.

### Ethics approval

All procedures outlined in this study protocol are in accordance with the 1964 Helsinki declaration and adhere to the ethical standards of: Children’s Health Queensland Human Research and Ethics Committee, Queensland, Australia (HREC/17/QRCH/159); Ethics Review Committee, Department of Human Services, Australia (MI8398) and Ethics Review Committee, Department of Education and Training, Queensland Government, Australia (550/27/1908). All families will give written consent to participate and they are able to withdraw their child from the study at any time without explanation or penalty from the research team and staff at Caboolture Hospital and Gold Coast University Hospital. Any protocol amendments will be submitted to Children’s Health Queensland Human Research and Ethics Committee, Queensland, Australia for review, as required. Any major changes to the study protocol will also be updated and reflected on ANZCTR.

### Trial status

This study is ongoing, with recruitment commenced in October 2017 and planned to continue for an 18 months period. It is planned that trial results will be published in relevant peer-review journals at the conclusion of the trial.

## Discussion

In our paediatric clinics across hospital sites, it is anticipated that a majority of recruited participants will be under 8 years old. A limitation of this study protocol includes the reliance of parental reported outcome measures for child behavioural and quality of life measures, particularly for children under 8 years old. Nevertheless, obtaining information from multiple sources aside from the child in the assessment of behaviours for school-aged children has previously been recommended in reducing reporting bias [25]. Other studies have also suggested that parents are much better at reporting on their children’s externalising, as opposed to internalising behaviours [26]. There is also a large body of research that suggest that there is a low to moderate relationship between parent and teacher’s reports of the child’s behaviours [27, 28]. In most studies, parents tend to have much higher scores of children’s misbehaviours, as compared with, the teachers [29]. As a result, many studies on school-aged children include measures which require parents to report on their child’s perceived emotional and behavioural states. It must be noted; however, that the current study will also attempt to obtain some self-reported data from children over 8 years old via the Peds QOL [20] and the SUDS [30]. Self-reported data for children older than 8 years old is generally regarded as the minimum age for reliable completion of questionnaires, as most will have mastered the basics of reading, writing and arithmetic skills commensurate with year levels 3 to 4 [31]. Based on available literature, this study proposes to focus on data based on a combination of parental and child self-reporting, dependent on the child’s age, developmental and cognitive levels.

In Australia, a National Disability Insurance Scheme (NDIS) [32] is being introduced progressively across the country from July 2016 onwards. The NDIS aims to provide support to all Australians under 65 years with a permanent disability to assist in planning their individual healthcare pathway [32]. A main component of the NDIS provides individuals with further information on their disability and referral options to existing support services available in the community. However, without strong empirical evidence and supporting frameworks, such an approach may provide significant challenges for families to navigate the health system to achieve appropriate coordinated and timely care for their child’s needs. It is anticipated that factors such as lower education, health literacy, socioeconomic status and knowledge of treatment/service options may impact on the successful uptake of available services via the NDIS [33–36]. Lack of resilience and networking may also impact on negotiating beauacratic road blocks and mis-information about to access care. Thankfully, the role out of the NDIS will not impact on the recruitment of participants for this study, as implementation dates at both sites for NDIS occur after this study’s planned recruitment timeframe: July 2018 (Gold Coast) and January 2019 (Moreton Bay, Caboolture), respectively. Nevertheless, outcomes from this study will help provide Australian healthcare system providers and policy makers on which types of families can successfully navigate the healthcare system themselves and which families need additional support. In particular, key resources and activities utilised within the ICCC pathway which provide the greatest health outcomes for participants in study may help guide care coordination frameworks for children with developmental chronic health conditions.

## Additional file

Additional file 1: Caregiver Information Sheet. (DOCX 406 kb)

## Abbreviations

ADHD: Attention deficit hyperactivity disorder
AHLO: Allied health liaison officer
aROC: Area under receiver operating curve
ASD: Autism spectrum disorder
CBCL: Child behaviour checklist
CP: Cerebral palsy
FASD: Fetal alcohol spectrum disorder
GCP: Good clinical practice
GCUH: Gold coast university hospital
GP: General practitioner
HREC: Human research ethics committee
ICCC: Integrated children’s clinic care
II: Intellectual impairment
NHMRC: National health and medical research council
ODD: Oppositional defiance disorder
PedQOL: Pediatric quality of life
SEIFA: Socio economic index for area
SLI: Specific language impairment
SUDS: Subject units of distress scale
