# Supplementary material for: Integrated children’s clinic care (ICCC) versus a self-directed care pathway for children with a chronic health condition: a multi-centre randomised controlled trial study protocol
Source: BMC Pediatr. 2018 Feb 19;18:72. doi: 10.1186/s12887-018-1034-x (PMC5817718; doi:10.1186/s12887-018-1034-x)
Supplement: Supplementary file 1 — Caregiver Information Sheet. (DOCX 406 kb) [file 12887_2018_1034_MOESM1_ESM.docx]

**Parent Information Sheet**

**HREC Project Number:** HREC/17/QRCH/159

**Research Project Title:** Healthcare Pathways for Children with Chronic Conditions

**Research Team:** Dr Thuy Frakking, Dr John Waugh, Dr Hsien-Jin Teoh, Dr Kelly Weir, Dr Doug Shelton, A/Prof Susan Maloney, Ms Donna Ward, Dr Michael David, Dr Matthew Barber, Dr Hannah Carter, Professor Sharon Mickan

**Version Number:** 3 **Version Date:** 10/08/17

**Thank you for taking the time to read this Parent/Guardian Information Statement and Consent Form. We would like to ask your child to participate in a research project that is explained below.**

**It is ok to say no**

**What is an Information Statement?**

These pages tell you about the research project. It explains to you clearly and openly all the steps and procedures of the project. The information is to help you decide whether or not you would like your child to take part in the research. Please read this Information Statement carefully.

Before you decide if you want your child to take part or not, you can ask us any questions you have about the project. You may want to talk about the project with your family, friends or health care worker.

**Important things to know**

- It is your choice whether or not your child can take part in the research. You do not have to agree if you do not want to.
- If you decide you do not want your child to take part, it will not affect the treatment and care your child receives through Children’s Health Queensland

If you would like your child to take part in the research project, please sign the consent form provided by the Researcher. By signing the consent form you are telling us that you:

- understand what you have read
- had a chance to ask questions and received satisfactory answers
- consent to your child taking part in the project

We will give you a copy of this information and consent form to keep.

1. **What is the purpose of this study?**

Children with chronic conditions require ongoing access to support across the school, community, hospital and GP sectors. However, long wait lists to access specialist services, limited health care funding and a shortage of appropriate health professionals within our community are barriers to fast and effective treatment for children with a chronic health condition. As a result, children with a chronic condition are at increased risk of mental health issues and social difficulties while awaiting support for their condition.

Care coordination, which has a central focus on child and caregiver needs, has been effectively applied to adult chronic conditions (such as diabetes, heart and lung disease) to improve quality of life, clinical outcomes and reduce costs to the health system. Care coordination has successfully been used in the management of asthma in children, but has not been applied to other chronic health conditions in children.

Our trial is aiming to find out whether access to a dedicated allied health professional results in better care coordination and health outcomes for children newly diagnosed with a chronic condition.

1. **Who is funding this research project?**

This study is being funded by a Queensland Health Allied Health Professions of Queensland (AHPOQ) Experienced Researcher Grant. Support is also being provided by the Allied Health and Paediatric Departments at Gold Coast University Hospital and Caboolture Hospital.

1. **Why have I been chosen to take part in this study?**

Your child is being asked to take part in this research study because they have been newly diagnosed with a chronic health condition by a Paediatrician and may be able to partake in the study.

Who can take part in this study?

- Children aged between 0 to 16 years with a newly diagnosed chronic health condition
- Written informed consent must be obtained from a caregiver

*Who will be unable to take part in this study?*

- Children with acute medical conditions requiring urgent attention from a medical specialist
- Children with a chronic health condition where medical management is primarily required. This includes cystic fibrosis, asthma, epilepsy.
- Children with a chronic health condition who are eligible for healthcare coordination within a hospital setting.

1. **What does the study involve?**

If you agree to be in the study, the researcher will assign your child into one of two pathways:

- - Pathway 1 – Integrated Children’s Clinic Care (ICCC)
  - Pathway 2 – Self-directed Care

This will be done by a process called randomisation, similar to tossing a coin, so you have an equal chance of being in either group. Neither you nor your researcher can choose which pathway your child is put in.

All caregivers will be asked to fill in questionnaires either in person or via phone at 1 week, 3 months, 6 months and 12 months after your appointment with the Paediatrician. The questionnaires will take about 1 hour or so to complete. Depending on your allocated pathway, appointments with GPs, schools, service providers may also be arranged for your child to help manage their chronic condition.

For all children involved in the study, the research team will access Medicare Australia records to obtain attendance records for GP, specialist and/or hospital visits during the time your child is in the study. You will be asked to fill out a consent form authorising the study access to your complete Medicare and Pharmaceutical Benefits Scheme (PBS) data as outlined on the back of the consent form. Medicare collects information on your doctor visits and the associated costs, while the PBS collects information on the prescription medications you have filled at pharmacies. The consent form is sent securely to the Department of Human Services who holds this information confidentially.

In addition, the research team will contact Education Queensland to obtain attendance records for the duration of your child’s time in the study. This information will help us to better understand overall costs to the healthcare system for the period of time your child is a part of this research project.

1. **Do I have to take part?**

You and your child do not have to take part in this study. We will describe the study and go through this information sheet with you and we will then give the information sheet to you. You are free to discuss the study with your family, friends or health care provider if you wish and to ask any questions. If you are able to help us with this study we will then ask you to sign a consent form to show that you have agreed for your child to take part. A copy of the signed informed consent form will be given to you. You are free to withdraw your child at any time, without giving a reason. This will not affect the standard of care your child might receive from any Queensland Health facility in the future.

1. **What if I wish to withdraw from the research project?**

Your decision whether or not for your child to participate will not prejudice their future relations with Queensland Health. If you decide for your child to participate, you are free to withdraw your consent and to discontinue participation at any time. The decision to withdraw from the study will not affect your child’s routine medical treatment or their relationship with the people treating them.

1. **What are the possible benefits for my child or me?**

Depending on which group your child is participating in, you may have an increased understanding of services offered in your area for the management of your child’s chronic condition. You may also have access to an additional healthcare professional that can help coordinate care across hospital, GP, community and school settings.

1. **What are the benefits for other people in the future?**

Depending on the final study results, children newly diagnosed with chronic conditions may have improved quality of life through faster and better linkages to ongoing care of their condition in the community. This may help to ensure that no children are ‘lost to follow-up’ and access support across community, GP, school and hospital sectors effectively.

1. **Alternative Treatment**

After a child is diagnosed with a chronic health condition by a Paediatrician, the usual standard of care relies on you, as the caregiver to coordinate further appointments with your child’s GP and other professionals involved in the school and community setting. This is also known as the “self-directed care pathway” of this research project. This standard of care is currently available to all caregivers of children diagnosed with a chronic health condition.

Alternatively, after a child is diagnosed with a chronic health condition by a Paediatrician, an alternative care pathway allows you access to an additional healthcare professional that can help coordinate care across hospital, GP, community and school settings. This is also known as the “integrated children’s care clinic pathway” of this research project. This alternative care pathway is currently not available to caregivers in usual clinical care, unless they are a part of this research project.

1. **What are the possible risks/discomforts for my child?**

There are no anticipated risks or discomforts for your child.

1. **Payments for your time spent taking part in this study or expenses:**

We do not anticipate there will be any cost to you or your child as a result of taking part in this study. You and your child will not be paid for taking part in this study.

1. **What will be done to make sure the information is confidential?**

Any information we collect for this research project that can identify you will be treated as confidential. We can share the information only with your permission, except as required by law.

Information will be stored securely within the Research Development Unit at Caboolture Hospital and within the Allied Health Sciences Department at Griffith University, Gold Coast Campus.

The following people may access information collected as part of this study:

- The research team involved with this project
- Relevant Human Research Ethics Committee

The information will be re-identifiable. This means that we will remove your child’s name and give the information a special code number. Only the research team can match your name to your code number, if it is necessary to do so. We will keep the information for 7 years. After this time, it will be destroyed.

The research data obtained from this research project may be published; however, identifying data will not be used. This means that your child’s name and individual clinical information will not appear on any publications. Reporting of any data for publication will be described as an entire group and/or described without any identifiable information about your child.

1. **Payments for your time spent taking part in this study or expenses:**

You and your child will not be paid for your time in this study.

1. **Will I be informed of the results when the research project is finished?**

On your request, we will send you a summary of the overall project results. The summary will be of the whole group of participants, not your individual results.

1. **What if I have other questions?**

If you would like more information about the project or if you need to speak to a member of the research team in an emergency please contact:

Name: Dr Thuy Frakking

Address: Research Development Unit

Caboolture Hospital

McKean St,

Caboolture QLD 4510.

Contact: (07) 5316 5972 | [thuy.frakking@health.qld.gov.au](mailto:thuy.frakking@health.qld.gov.au)

| The Children’s Health Queensland Hospital and Health Service Human Research Ethics Committee (HREC) has approved this study. If you have any concerns and/or complaints about the project, the way it is being conducted or your child’s rights as a research participant, and would like to speak to someone independent of the project, please contact the HREC Coordinator on:  3069 7002 or email [CHQETHICS@health.qld.gov.au](mailto:CHQETHICS@health.qld.gov.au) |
| --- |
